# Supplementary material for: Rethinking success, integrity, and culture in research (part 1) — a multi-actor qualitative study on success in science
Source: Res Integr Peer Rev. 2021 Jan 14;6:1. doi: 10.1186/s41073-020-00104-0 (PMC7807516; doi:10.1186/s41073-020-00104-0)
Supplement: Supplementary file 1 — Additional file 1. [file 41073_2020_104_MOESM1_ESM.pdf]

Supplementary file

Rethinking success, integrity, and culture in research (part 1) — A multi-actor qualitative study on success in science

Noémie Aubert Bonn, Wim Pinxten

Table of contents

1. RESEARCH TEAM AND REFLEXIVITY 2

2. COREQ CHECKLIST 4

3. GENERAL INTERVIEW GUIDE 6

4. GENERAL FOCUS GROUP GUIDE 8

5. SAMPLE QUOTES ON PUBLICATIONS 11

6. SAMPLE QUOTES ON THE IMPACT FACTOR 12

7. SAMPLE QUOTES ‘WISH FOR CHANGE’ 13

1. RESEARCH TEAM AND REFLEXIVITY

In accordance with the COnsolidated criteria for REporting Qualitative research checklist (COREQ; page 4), and in respect of transparency, we found important to provide further characteristics about the setting and the interviewer at the time of the study.

Besides one early interview with an institution leader in which WP, assistant professor, attended to provide feedback about the interview, all other interviews and focus groups were conducted by NAB, with no additional non-participant or assistant.

NAB is a female PhD student in the Faculty of medicine and life science of Hasselt University, Belgium, with a background in cognitive neuroscience and bioethics. Coming from Canada, NAB had the advantage of bringing a certain neutrality in the interviews by not being strongly affiliated with one or another Flemish region, and by not corresponding to an established research group.

Before conducting the interviews and focus groups, NAB followed courses about developing interview questions, conducting focus groups, and analysing qualitative data offered from Flemish universities and from the Flanders' Training Network for Methodology and Statistics (FLAMES). In addition, she used the resource books from the Focus Group Kit by Richard A. Krueger and David L. Morgan (Morgan & Krueger, 1998), and discussed with RDV — expert in qualitative inquiries and part of the team that built the original guide upon which we inspired ours — to gain insight on building, conducting, and analysing focus groups and interviews.

Besides a few exceptions, NAB had no prior relationship with most participants, and the first contacts were established with the invitation email. No repeat interviews were carried out. Before the interview, NAB described the project briefly and explained the purpose of the interview informally. On some occasions where interviewees were anxious to know more about the project in advance, NAB would email the main themes targeted, but would not share the interview guide with participants by fear that this may lead to rote answers.

Bias and assumptions

NAB holds the view that research integrity is largely determined by the research system, and the interview guide was necessarily not unbiased to this perspective. Nonetheless, if participants shared a different view (e.g., if they believed that integrity was solely a matter of personality), NAB was careful not to contradict or bias interviewees' ideas towards her perspective. In re-reading quotes with the research team, we were careful for possible misinterpretations, and when quotes were interpreted differently by WP or RDV, we adapted the nodes and interpretations to make sure they fit the words of the participants. Both WP and RDV helped in classifying the main nodes into general categories of *Who*, *What*, *How*, and *Luck*. Initially, we were tempted to classify these four categories in *Products* of success (the What) and *Potential* for achieving success (the Who, How, and luck). However, after several discussions, we realized that doing so may reinforce the perspective that products are the ones which truly indicate success, while potentials are simply increasing the chance of yielding better products. As we describe in our extended findings, many of our interviewees considered the *Who* and especially the *How* to be genuine successes in themselves. In this regard, we intentionally kept the four categories together as each representing successes in themselves.

Study design and interview/focus group setting

Interviews and focus groups were conducted in private meeting rooms or offices or, according to preference, in public spaces (N=2) or through video call (N=3). One of the interview conducted through video call had some sound and connection problems, but the other video calls went very smoothly.

Interviews lasted on average 60 minutes, depending on the time granted by the interviewee (range from 34 to 80 minutes). Focus groups lasted around 120 minutes each and included a five-minute break.

All interviews were audio recorded and transcribed verbatim by the interviewer (NAB) or a university-approved transcription service. Transcripts were not returned to participants except in select cases where participants expressed a wish to monitor their answers, and in cases where the quotes of interest might have jeopardized the confidentiality of participants. No repeat interviews were undertaken. After most interviews, the interviewer filled a self-questionnaire about the interview to note any abnormalities and general feelings of the interview data.

Reference

1. Morgan, D. L., & Krueger, R. A. (1998). London: Sage Publications.

2. COREQ CHECKLIST

COnsolidated criteria for REporting Qualitative research

Topic	Item No.	Guide Questions/Description	Reported in section:
**Domain 1: Research team and reflexivity**			
Personal characteristics			
Interviewer/facilitator	1	Which author/s conducted the interview or focus group?	Supp. file
Credentials	2	What were the researcher's credentials? E.g. PhD, MD	Supp. file
Occupation	3	What was their occupation at the time of the study?	Supp. file
Gender	4	Was the researcher male or female?	Supp. file
Experience and training	5	What experience or training did the researcher have?	Supp. file
Relationship with participants			
Relationship established	6	Was a relationship established prior to study commencement?	Supp. file
Participant knowledge of the interviewer	7	What did the participants know about the researcher? e.g. personal goals, reasons for doing the research	Supp. file
Interviewer characteristics	8	What characteristics were reported about the inter viewer/facilitator? e.g. Bias, assumptions, reasons and interests in the research topic	Supp. file
**Domain 2: Study design**			
Theoretical framework			
Methodological orientation and theory	9	What methodological orientation was stated to underpin the study? e.g. grounded theory, discourse analysis, ethnography, phenomenology, content analysis	'Methods' → 'Analysis'
Participant selection			
Sampling	10	How were participants selected? e.g. purposive, convenience, consecutive, snowball	'Methods' → 'Participants'
Method of approach	11	How were participants approached? e.g. face-to-face, telephone, mail, email	Supp. file
Sample size	12	How many participants were in the study?	Table 1
Non-participation	13	How many people refused to participate or dropped out? Reasons?	—
Setting			
Setting of data collection	14	Where was the data collected? e.g. home, clinic, workplace	Supp. file
Presence of non-participants	15	Was anyone else present besides the participants and researchers?	Supp. file
Description of sample	16	What are the important characteristics of the sample? e.g. demographic data, date	Table 1
Data collection			
Interview guide	17	Were questions, prompts, guides provided by the authors? Was it pilot tested?	Supp. file
Repeat interviews	18	Were repeat inter views carried out? If yes, how many?	Supp. file

Audio/visual recording	19	Did the research use audio or visual recording to collect the data?	Supp. file
Field notes	20	Were field notes made during and/or after the interview or focus group?	Supp. file
Duration	21	What was the duration of the inter views or focus group?	Supp. file
Data saturation	22	Was data saturation discussed?	_
Transcripts returned	23	Were transcripts returned to participants for comment and/or correction?	Supp. file
**Domain 3: analysis and findings**			
Data analysis			
Number of data coders	24	How many data coders coded the data?	Page 7-8
Description of the coding tree	25	Did authors provide a description of the coding tree?	Figure 1 and Figure 3 in (1)
Derivation of themes	26	Were themes identified in advance or derived from the data?	'Methods' → 'Analysis' and Supp. file
Software	27	What software, if applicable, was used to manage the data?	'Methods' → 'Analysis'
Participant checking	28	Did participants provide feedback on the findings?	Supp. file
Reporting			
Quotations presented	29	Were participant quotations presented to illustrate the themes/findings? Was each quotation identified? e.g. participant number	Throughout
Data and findings consistent	30	Was there consistency between the data presented and the findings?	Throughout
Clarity of major themes	31	Were major themes clearly presented in the findings?	Figure 1 and Figure 3 in (1)
Clarity of minor themes	32	Is there a description of diverse cases or discussion of minor themes?	Throughout

Reference

1. Aubert Bonn N, Pinxten W. Rethinking success, integrity, and culture in research (part 2) – A multi-actor qualitative study on problems of science. Research Integrity and Peer Review. 2020.

3. GENERAL INTERVIEW GUIDE

A part of my research is to explore the views of different actors that contribute to the research system.

To protect your privacy, I want to avoid disclosing your specific job title and to place you in one of bigger category of research actors. I may use a higher level of details to describe the type of participants included in each category, but I won't link direct quotes with company or institution names.

I placed you in the category ***actor group***. Does that sound good to you?

Introduction and information on respondent's career

1. Before anything, I would like you to **describe your work** to me, in your own words.

   Prompt: In a broader perspective, what would you say is your role is in the scientific system?

2. In this job, you obviously **care for scientific excellence**. How would you say you fulfil this goal in your work?

   We will get back to this a bit later. For now, I will change topic and I want us to talk about success as this is an important topic that we are trying to understand in the project.

Success in science

3. First, try to think about scientists you've known that were very successful. What do you think made these scientists **successful**?

   Prompt: Which characteristics do you think are most important to advance a researcher's career?

4. Do you feel like these characteristics are **captured in current research assessments** and evaluations? In which ways?
5. (If time allows) What do you feel that your actor group **should do to promote successful science**? Do you see that happening?

Tensions or conflict between success and integrity

6. You mentioned that X, Y, Z are criterions that indicate success in research. Do you think that these are **also indicators of quality**? Sound research?

   *Prompt: Which criterions do you think **indicate the quality** of the research?*
   *Prompt: Which criterions do you feel are **not suited** to indicate the quality of the research? Explain.*

7. Does it happen that you see excellent researchers but for some reason these researchers **don't succeed in getting ahead** with their career? Can you give me some examples?
8. Do you feel that the way in which success is attributed allows to for **emerging scientists** to become successful?

Current problems

Let's change the topic now; leave aside success for a bit and look at when science is not at its best. Like I said, I am not here to denounce or condemn cases, so I will make sure to protect the confidentiality of cases you may discuss.

9. Have you ever had to **deal with science** which you considered was **not really in line with the rules of science**? What happened?

10. Can you give me precise examples of the elements that you consider **signs of bad or sloppy research**? What are **red flags**?

Motives for bad practices

11. **Why** do you think bad research practice happens?
12. Do you think **anyone could** end up in such a situation or only types of people?

Responsibilities towards integrity

We have already discussed how to promote successful science, now I would like to gather your thoughts on how to prevent sloppy research.

13. What do you think should be done to **prevent bad science** from happening?
14. **Who** should take the lead to make these changes happen? Who else should be involved?
15. What do you consider is the **responsibility of your *actor group*** to protect integrity?
16. Where does your responsibility **end**?

One change

Finally, if you could pick **one important change that needs priority** right now in how research works, what would it be? How do you think this change could be done?

(if time allows) Alternatives

17. If there were no rules for evaluating scientists, and you could **start from scratch**, what would you like to look at when assessing scientists?

> *Prompt: What are the characteristics that **YOU think are most important** for researchers to do good research?*

4. GENERAL FOCUS GROUP GUIDE

Intro:

1. Who you are
2. What is your area of research
3. Describe a typical day of work
4. What's your favourite ice cream flavor

Scientific career

Before starting, I would like to know a little more about your career as a researcher.

18. Specifically, I would like to know what is it that makes your work so great? What do you feel is most satisfying, most rewarding about your career?

    Prompt: When people ask you why you chose to be a researcher, what first comes to mind?

We will get back to this a bit later. But for now I will change topic and I want us to talk about success.

Success in science

19. Think of a person in your field who you think is **very successful**. (It doesn't have to be one person in particular, it can just be some characteristics of many different people, can be yourself in 20 years...) How do researchers **become successful**? What, in your view makes this person a success?

    Prompt: What are the most important factors for advancing in your career?
    Prompt: What are the funders and the employers looking at?

20. Now imagine that I am a newcomer in your field and I ask you what I must do to stay on the top, **what would you tell me**?

So you say that successful scientists are generally scientists who do X, Y, Z.

21. Do these successful scientists **reflect or mirror the kind of scientist you want to be**? Do you have such aspirations for success?

Tensions or conflict between success and integrity

22. As we discussed, you point out that funders and employers look at X, Y, Z... Do you think that **these criterions for success indicate outstanding or excellent research** (e.g., appropriate methods, relevant topic, high quality work)?

    Prompt: Which criterions do you think ***indicate the quality*** *of the research?*
    Prompt: Which criterions do you feel are ***not suited to indicate the quality*** *of the research? Explain.*

23. Now try to think of a **colleague** who, in your opinion, **does good research but cannot reach success in science**?

    Prompt: *What in your opinion explains that this researcher cannot reach a successful career?*

24. **What would you say to this researcher to help him/her get ahead**?

Current problems

Let's change back the topic now; leave success aside for a bit and discuss what it is like to be a researcher. So you remember that at the beginning of the discussion, I asked you about the aspects of research that make you like your career. Now I want us to talk about the other side of things, about what **frustrates** you as a researcher.

25. So let's say I am a **newcomer** in your field. I just started working in your lab and I am not sure whether I should follow a scientific career. If I asked you what are the **most frustrating things about working in science**, what would you tell me?

    Prompt: what would you tell me are some of the biggest frustration I could encounter?

26. All right, so as we have discussed, being a researcher is not necessarily always easy. It can sometimes happen that things go really wrong. Have you **ever seen or heard** of a situation in which you thought research was conducted in a way that was **against the 'rules' of science'**? What happened? What did/would you do?

Motives for bad practices

27. **Why** do you think researchers were acting in this way?
28. Do you think **any researcher could end up** in such a situation?

CURRENT VIEWS ON RESPONSIBILITY

29. **What** do you think should be done to **prevent bad science** from happening?
30. **Who should take the lead** to make these changes happen? Who else should be involved?
31. **What can you do**?

    Prompt: Do you feel like you miss something to be able to change things yourself?

Solutions

To finish, I would like to ask a more concrete question.

32. Finally, if you could pick **one important change that needs priority** right now in the research system, in how science works, what would it be?

    Prompt: How do you think this change could be done?

Personal success

Now before we finish, I want you to think back about the discussion we have had on success, and on criterions that are most often used to evaluate a research career. But now, I would like you to think about yourself as a researcher, and to think about your strength, about what makes you feel accomplished in your work. **What do you think is your biggest contribution to your work**, or **something that you think is key to be a good researcher**, regardless of the criterions we have said before. (For example, maybe you think that the fact that you brush your teeth after lunch is key to the success of your research team.)

I will not ask you to discuss it this time, but I would like everyone to take one of these little pieces of paper. On the piece of paper, I would like you to make up 3 to 5 criterions for funders and employers. I want you to think about what you consider your biggest contributions in your work, and to make up criterions you would think, if funders and employers evaluated, you would have better chances to succeed.

Summary

Is there anything you would like to mention that we failed to discuss today?

5. SAMPLE QUOTES ON PUBLICATIONS

Sample quotes substantiating arguments against and for using publications as a main determinant for scientific success.

Argument	Sample quote	actor
**Arguments against using publications as the main determinant of scientific success**		
Reductionist	*It is a very flawed measure of success in a way. I mean… I don't want to give the impression that… of discouraging any of these successes, you know, I mean publishing very important papers in very selective journals is an achievement, that is very clear. But I think that there are other very important contributions to the scientific enterprise which don't necessarily translate into one of these unit of credit of success, which is a first author publication in a very prestigious journal. And I think that currently we collectively, as a community, do not do enough to actually support and reward these kinds of contributions that are very important for the scientific enterprise*	EP
Arbitrary	*Yeah but with publications it's sometimes also just having luck…[…] To me it's not always that you're a good researcher.*	LT
	It is wrong to think that... […] having more publications, it means you're better and better and better, I think it's a very wrong way of thinking.	PMI
	I have less and less confidence in publishing with the fact that 'who is going to be the reviewer?' 'Is he biased?' 'Is it the journal?'	R
	The highest journal [of my field] it's all already fixed before with companies, pharmaceutical companies, who will get published their RCTs, it's already all set in advance…	PhD
Perverse	*They do a lot of experiments just to publish. Just to make an article, because they have to have an article before the four years are done. So they do their experiments in function of an article*	LT
	It's my only drive for some things, that it's just publication.	Res.
**Arguments in favour of using publications as the main determinant of scientific success**		
Representative	*So people say, you know, publications don't matter, but at the end of the day there clearly is a link. If you end up publishing in a good journal, then you probably started off with a very good research question, and you probably are a very good researcher. They are not 100% linked, but I'm sure there is a link there.*	RIL
Measurable	*"It's the career, it's the way you get the career, it's the number of publications that will count, the number of promotions of PhD theses will count, but for me that's not the most important. I think a researcher who is not... who is publishing (they need to publish of course) but let's say only two A1 publication, or one publication a year, but in the meantime is contaminating other researchers, helping other researchers and is multidisciplinary... That's more valuable for me as a person. But in the academic world,* ***I cannot value that directly****. I'm not in a position that I can say "You are the very best researcher, so I promote you to full professor from associate professor". Because there we still have the numbers that count. And ok, that's the way it is, and that's the whole issue nowadays with researchers. They really get troubled with these numbers."*	RIL
	"I think it would also be a bit difficult to really value a PhD or the PhD project without publications. Because how do you determine that someone has done their best, but unfortunately didn't get any publications."	PhD
	"I do believe that you have to have some evidence about the process you have made, and the path that you've walked throughout your doctoral thesis. That's why I find it quite normal that you have to have a certain amount of publications in the procedure… "	RIO
Necessary	*"I think I'm going to be the boring one, but I think it is important to have publications and to also be successful in some research grounds every now and then because I feel like it's my... That's what is expected from me, but that's also how you can make the research... you can keep the research going. I think it's one I see as my duty to publish the results, to share them so that others can build further on them and you yourself can build further on them."*	Res.
	"If you don't have the publications you're not noticed. And if you're not noticed, your research might be extremely interesting, but if it's not read, if it's not noticed, what's the value."	RCC

Note: Researcher is abbreviated to Res.

6. SAMPLE QUOTES ON THE IMPACT FACTOR

Select quotes exemplifying the disagreement around the use of impact factors in research assessments.

Sample quote	actor
**SELECT QUOTES IN FAVOUR OF USING THE IMPACT FACTOR**	
**Several interviewees perceived the impact factor as a measure of quality of a journal**	
"So if you hire a PhD student, but even more if you hire a postdoc or a young professor, then they evaluate it of course. And then, the bibliometric parameters are much more important so you look at the number and the quality of publications. ***How do you measure the quality, of course the impact factor****. So if somebody with a Nature paper comes of course this person is considered to be more 'valuable', in quotation marks, and gets a higher score in the end and probably the job, compared to a person with a publication record which has lower impact factors. So impact factors are still very important, and grants..."*	*RIL*
"Of course what you always want to have is one of the two champions that are really picky in the graph, but I think for us it's also important to really see that the whole group is evolving to improved ***quality as measured by the impact factor*** *and of course I know the discussion that this is only one way to look at quality, but* ***it's still the most accepted way to look at quality*** *I think, in our field."*	*RIL*
"I have to say that generally there is a ***big correlation between the impact factor and the quality*** *of the content…"*	*EP*
"OK, when we select something, somebody for an academic position, we will look at publications, at the numbers, and below 10 you will never get something in an academic position, below 10 papers. Of course, suppose somebody comes with two Nature, one Lancet, and one NEJM, then we have to re-think. So... In a way, today it's still a balance between numbers and impact factors, it's still playing a role. But the whole issue is that there is something which goes together. ***A journal with a high impact factor has to improve its review process.*** *Because you cannot keep your high impact... I think that when you send your paper to Lancet or NEJM, you will have tough review. While when you send it to a low impact factor journal, […] you can send a completely fake paper to reviewers who will judge it perfect and let it publish."*	*RIL*
**SELECT QUOTES AGAINST USING THE IMPACT FACTOR**	
**Most participants however, argued against the use of impact factors, stating that they were irrelevant for individual paper assessments, that they disadvantaged fields of research, and also that they were simply not an indicator of quality**	
"I think [current metrics are] ***far too simple****. You know like impact factor is* ***useless I think in evaluating the importance of an individual paper****, because impact factor relates to a journal. So it's not an article level measure of any kind."*	*EP*
"Publishing is important but I hate the impact factor thing. I would more look into the quartile thing, if you are ***in a field that has low impact factors*** *but you are in the top ten of your field, that's just fine. I mean it doesn't have to be Nature, it can also be [a small specific journal], if that's your top, in your field. So I think there is a tendency towards going that way but I like that a lot more than the impact factor shizzle, yuck!"*	*RCC*
"Interviewer: *Which [indicators] do you think are the most toxic and less representative of quality?*<br>Participant: *The urge to publish in Q1. […] I understand that there needs to be an impact factor, but the whole issue of the weight of an impact factor in the personal career of a researcher… because then I would advise anybody who wants to go in research "Please go in cancer research". Try to get to Lancet cancer or whatever other journal, of NEJM and then you're safe. Don't do anything like plastic surgery or [smaller topics]... So* ***that's one of the most toxic factors I think****. The pressure of... Because the* ***impact factor is not reflecting really the importance of the research****. You could say that cancer is of course important, and then you see that for instance [the biggest journals in other discipline] which has an impact factor of 16, they only publish on cancer [...] and they* ***manipulate the impact factor****. Of course, because when you, as an author, you don't have enough references referring to their own journal you get from the reviewer report that you need to put those in..." (RIL, bold added for emphasis)*	*RIL*
"Well the problem with the impact factor as a standard, most appreciated metrics, even though we don't want to do that [laughs], is that it is ***not essentially an indicator of quality neither of the article, neither of the journal****, but why? Because there could be less articles of lesser quality, published by renowned scientists in higher impact factor journals, and you can have a good research from scientists coming from some small country and who is not so famous internationally, and he will not, or she will not be able to publish in the higher impact factor journals because they are usually biased, and I know because I come from a country, when you read someone's last name you usually… they can know that you are from that country [laughs]."*	*EP*

7. SAMPLE QUOTES 'WISH FOR CHANGE'

Sample quotes from the 'wish for change' which relate to changes in the ways success is defined and assessed.

Actor	Sample quote
**CHANGES TO RESEARCH ASSESSMENTS**	
Value quality over quantity	
RIO	*I will then insist that the money is spent on projects of high quality. Quality of the research.*
Res.	(immediate response) *Take the output pressure away! So you can have more room for quality.*
PMI	Participant: *My wish is that scientific outcomes, papers, pieces, news, are assessed by their intrinsic value, intrinsic scholarly value and not by indirect measures as it is the case right now. Journal impact factors, citation index, et cetera, these are all proxies.*<br>Interviewer: *What would you say is any intrinsic value?*<br>Participant: *I think open peer review would solve this problem.*
Reduce output pressure and competition	
EP	*I would like to see a world where the pressure is off the researchers, you know, not... there are not pressured, in the world that they can do their research without pressure of publishing in high impact journals, and like to see that there is no impact factor anymore at least not in such a way that there is usually considered today. And that to bring more joy in their life, essentially, because I think that they are so stressed out, and they are always chasing some next step in their career advancement, and they forgot that the science is actually fun thing to do, you know, it can be a way of, you know, living a life, not just working as a hamster in a wheel, you know, just yeah, chasing your own tail or something like that.*
EP	*(Laughs!) It is a really tough one. Because I don't see… Do you know Merton's model? […] OK. There is tension, there is obvious tension between the kudos, and the whole system that has been put in place where it's… you have to be special. It doesn't fit. It doesn't fit with the kudos! It doesn't fit with the universalism, etc. So I think that that's where something is wrong. I don't have the solution, but that's what needs to be addressed! [...] I would try to solve that tension that exists right there, to be able to go back to the other communalism, to the universalism etc. You know, the kudos itself.*
EP	*Change the reward system! (Laughs) Change the reward system. Completely. Because would then allow everyone A) to publish wherever it's really most relevant, it's not linked to the impact factor any longer… You know if people did that, what I said, and this was not relevant, impact factor was not relevant, and it's really truly about what kind of research career have I had and what research have I done, you know, that is really important, and how does this impact in my field. Then I think everything would change. And, yeah, that would be my biggest wish, and I'm working towards that.*
Broaden and adapt indicators	
PhD	*Maybe the cumulative impact factor that they just need to do it really field per field, and not faculty per faculty*
RIN	*I think it would be broadening of the criteria for recruitment, promotion, funding. I think if we could really get everybody behind that, it would have a huge impact I think.*
PhD	*...maybe looking at PhD as a career. Because now you have only one main outcome, the publications, but in a career you have a lot of competencies that are important.*
EP	*If I have a magic wand, I think I would want to get rid of the Impact Factor in research assessment. And getting rid of… You know changing this problem that we started this conversation with. Which is that it's only publication in a certain amount… in a small number of very selective journals that is considered a measure of success. So, you know, I would want a magic number that represents all these other things and that's probably, that's completely unrealistic, but I would want at a minimum the research assessment framework to change to move away from that single dominant measure that is being used at the moment. To appreciate different kinds of contributions much more effectively.*
**CHANGES TO RESEARCH FUNDING**	

Fairness in evaluation	
PhD	Participant A: *I think that... I feel that there is a bias that certain groups will always get funding, and smaller universities [...] are really struggling to get like an FWO project funded. So maybe there should be some regulations about it. But I don't know how... maybe restricting the number of projects that the group can apply for. I think they already have some regulations for that...*<br>Participant B: *I think so too.*<br>[...]<br>Participant C: *Anonymous selection... (laughs)*<br>Participant A: *That would be maybe the best.*<br>Participant B: *But that is also important, because a lot of professors or researchers knew people that are the judges, and they have like, the privilege, and will get funding. So anonymous would be better.*<br>Participant A: *But then maybe you lose the advantage of collaboration. You can't say "but we'll be collaborating".*
Fairness in distribution	
EP	*Yes, my fairy wish would be a change of the grant system, and I'm saying of that it… in my… so say 80 percent of the money might be divided according to the prevailing system, for the proposals and give 80 percent to the best proposals, and then we have a pile of proposals that are rejected, and make it a lottery, for 20 percent.*
RCC	Participant: *More freedom. Less bullshit. More... and maybe the money should be just divided equally or something like that, which is also not really realistic because then the amount would probably be so small that you still cannot do anything. But at least then everybody can not do anything, instead of being, you know, when you have this big in house thing and here is the people that get a lot of money and get a lot of stuff done, and here's me [laughs]. At least we all will be...*<br>Interviewer: *Everyone would be fair.*<br>Participant: *Yeah, it's just not fair. That's it, it's not fair. And if you... I can completely understand why big science people don't go to [small university] because you kill your career if you [go there].*
Long-term and baseline funding to increase security	
RIL	*I think it's the research funding, but I don't... I just know it should change, but I don't have the answer for you. I think a researcher should not have these short term financing situations. I think that's probably the worst perverse incentive you can give a scientist. I think you should have a Tenure track where you require that a scientist proves him or herself, but once you have an established scientist, they should have some sort of basic funding which could be adjusted based on how they perform, but it should not be this 'yes/no' thing on a four year term which is what most grants are. Because I really need to deliver in four years, and that gives me perverse incentives.*
RCC	*But maybe it might be interesting to give people different kinds of contracts. To don't give always these short-term contracts, but give people longer term contracts. But I know that there's a discussion. I know a lot of people say 'well I give the best of myself because I have a short-term contract and the edge is on… I don't know whether the edge should be so strong. I don't know whether the competition should be so strong. I don't know whether that's really helpful. If you really want to achieve trust and if you really want to achieve openness to mistakes, people should feel secure enough to do it. And I think one of the answers is 'you will not lose your job'. So… Maybe job security might be an answer. (RCC)*
FA	*Hm hm. Well exactly what I said form the start. I think that we should have a very close look at the way we are funding institutions for doing their research. I think this is key […] but there are some elements I recognise, and we recognise, that are worth a good discussion. And one of these elements is that indeed apart from competitive funding, which is important because competition, and what we are doing here can make for good quality research, excellent research, and apart from this competitive funding, you also need some sort of basic funding to give people a chance to start and to launch their career as an academic. Also to do some things that are less fashionable, because also research has its fashions, less fashionable, or less appealing to evaluators at the moment, with which you can prove after a while that there is something in it and then you become stronger to an evaluation panel. So I think that reconsidering the way you are funding research institutions is also letting some pressure, or diminishing some pressure on institutions like us. I think you get better competition, by also making it less stringent. Maybe this sounds as a paradox, but I don't think it is.*
Res	*Start-up money? For creative plans which are not judged from the beginning?*

Note: Researcher is abbreviated to Res.
